# Assessing the relationship between dietary factors and hair health: A systematic review

**DOI:** 10.1177/02601060251367206

**Published:** 2025-08-21

**Authors:** Nuno Gomes, Nuno Silva, Beatriz Teixeira

**Affiliations:** 1 Faculdade de Ciências da Nutrição e Alimentação, 72968 Universidade do Porto, Porto, Portugal; 2 EPIUnit ITR, Instituto de Saúde Pública da Universidade do Porto, 72968 Universidade do Porto, Porto, Portugal

**Keywords:** Alopecia, hair loss, hair quality, nutrition, systematic review

## Abstract

**Background:**

Specific foods and nutrients have a significant importance in maintaining healthy hair, which is a crucial aspect of an individual's identity and self-esteem.

**Aim:**

To identify, through a literature review, the association between the consumption of specific foods and/or nutrients and the health of the hair.

**Methods:**

This review followed the PRISMA guidelines and was registered on the PROSPERO platform (registration number: CRD42024527250). The databases Medline (PubMed), Web of Science and Scopus were consulted between March and June 2024. The following inclusion criteria were considered: individuals aged ≥3 years old; the consumption/intake of specific foods/nutrients; and articles written in English and Portuguese. The search expression combined terms related to ‘diet’, ‘nutrition’ and ‘hair health’ (*n* = 1287 articles), where language filters and duplicate removal were applied.

**Results:**

The analysis of 17 studies involved 61332 participants, predominantly women (97%). Vitamin D stood out as the most studied nutrient (five studies), while alopecia and hair loss were the most studied hair health parameters (eight and five studies, respectively). Higher levels of vitamin D and iron were inversely related to alopecia. Conversely, a higher intake of alcoholic and sugary beverages was found to be positively correlated with hair loss.

**Conclusion:**

Diet and nutrition play a crucial role in hair health, particularly vitamin D and iron supplementation, while limiting alcohol and soft drinks may be beneficial. Further research is needed to confirm these findings.

## Introduction

Hair is an important individual characteristic that plays a fundamental role in an individual's self-esteem and identity. In recent decades, there has been a growing interest in investigating the role of diet and nutrition in hair health (Gokce et al., 2022). While the precise mechanisms remain unclear, studies indicate that certain vitamins and minerals, such as vitamin D and iron, are essential for the normal development of hair follicles (Cruz et al., 2020; O'Connor and Goldberg, 2021; Rajput, 2022).

Similarly, it is evident that a deficiency in specific nutrients can contribute to the onset of alopecia (Almohanna et al., 2019). This condition is defined by the absence or reduction of hair in an area where it would normally be expected, affecting individuals across different age groups and genders (Al Aboud and Zito, 2024). From a clinical perspective, hair loss is a prevalent concern in dermatological practice, and it has a significant psychological and emotional impact on those affected (Aşkın et al., 2022).

The relationship between diet and hair health remains underexplored in the literature. Recent reviews (2021–2022) have primarily focused on the role of nutrients and alopecia/hair loss (Gokce et al., 2022; O'Connor and Goldberg, 2021; Rajput, 2022), highlighting the need for further investigation research in this area. However, few studies have examined the combined effects of foods, supplements, and nutrients on hair health. This study synthesizes the existing literature, providing a comprehensive overview of the association between diet, nutrition, and hair health. The findings may serve as a valuable resource for both individuals and healthcare professionals seeking to implement more effective interventions (Gupta et al., 2021; Hailemariam et al., 2019; McKibbon, 1998). This review aimed to identify associations between specific foods and/or nutrients and hair health based on existing literature. To meet this objective, the eligibility criteria for the studies were structured using the PICOS framework, which defines the population, indicator, comparator, outcome, and study design.

## Method

### Study design

This systematic review was conducted in accordance with the PRISMA methodology (Matthew et al., 2021) and registered prospectively in the PROSPERO software (International Prospective Register of Systematic Reviews) as CRD42024527250 (Gomes et al., 2025).

### Search strategy

The search expression was carried out and applied between March and June 2024 in three databases (Medline-PubMed, Web of Science and Scopus). The search expression entered into the Medline database was the following one : (‘energy deprivation’, ‘food restriction’ or starvation or malnutrition or ‘dietary intake’ or ‘nutritional status’ or (food consumption [MeSH Terms]) or ingestion or ‘dietary factors’ or (nutritional deficiencies[MeSH Terms]) or ‘diet quality’ or ‘restrictive eating’) AND (‘hair health’ or ‘hair quality’ or ‘hair growth’ or ‘hair follicles’ or ‘hair texture’ or ‘hair strength’ or ‘hair density’ or ‘scalp health’ or ‘hair shedding’ or ‘hair loss’ or ‘hair maintenance’ or ‘hair thinning’ or ‘hair damage’ or ‘hair color’). A comparable search expression was employed in the Scopus and Web of Science databases. The search expression was conducted by the principal investigator and subsequently reviewed by the remaining members of the research team.

### Eligibility criteria

The eligibility criteria employed to select the studies are detailed in Table 1, in accordance with the PICOS criteria. This review considers only studies written in English and Portuguese.

**Table 1.** Eligibility criteria of the included studies, according to population, indicator, comparator, outcome, and study type (PICOS).

PICOS	Inclusion criteria	Exclusion criteria
Population	Individuals aged ≥ 3 years old.	Individuals aged < 3 years old; Previous diagnosis of special dietary needs; Studies not conducted on humans.
Indicator	Food and nutrients assessed individually through for example food diaries and food frequency questionnaires; Nutritional supplementation.	Dietary patterns rather than individual foods or nutrients.
Comparator	Not applicable.	Not applicable.
Outcome	Hair growth, quality, texture, health, strength, and maintenance.	Hair growth, quality, texture, health, strength, and maintenance, but not associated with specific foods or nutrients.
Study type	Original studies.	Reviews, abstracts only, case studies of one individual, books, and conference papers.

### Study selection and data extraction

A total of 1287 articles were obtained from the three databases using the specified search expression. By applying a search filter (language filter: Portuguese and English) and removing duplicates, a final set of 880 articles was obtained. Two independent reviewers screened the titles and abstracts of these articles based on predefined inclusion criteria In case of disagreement regarding article inclusion, a third researcher was consulted to facilitate discussion and assist in the decision-making process. The full texts were then independently assessed by the two reviewers, with the third researcher providing guidance and resolving discrepancies when necessary. After finalizing the inclusion and exclusion criteria, a comprehensive analysis of the selected references was conducted using the snowballing technique *(Wohlin, 2014)* to identify additional relevant studies

**Figure 1.**
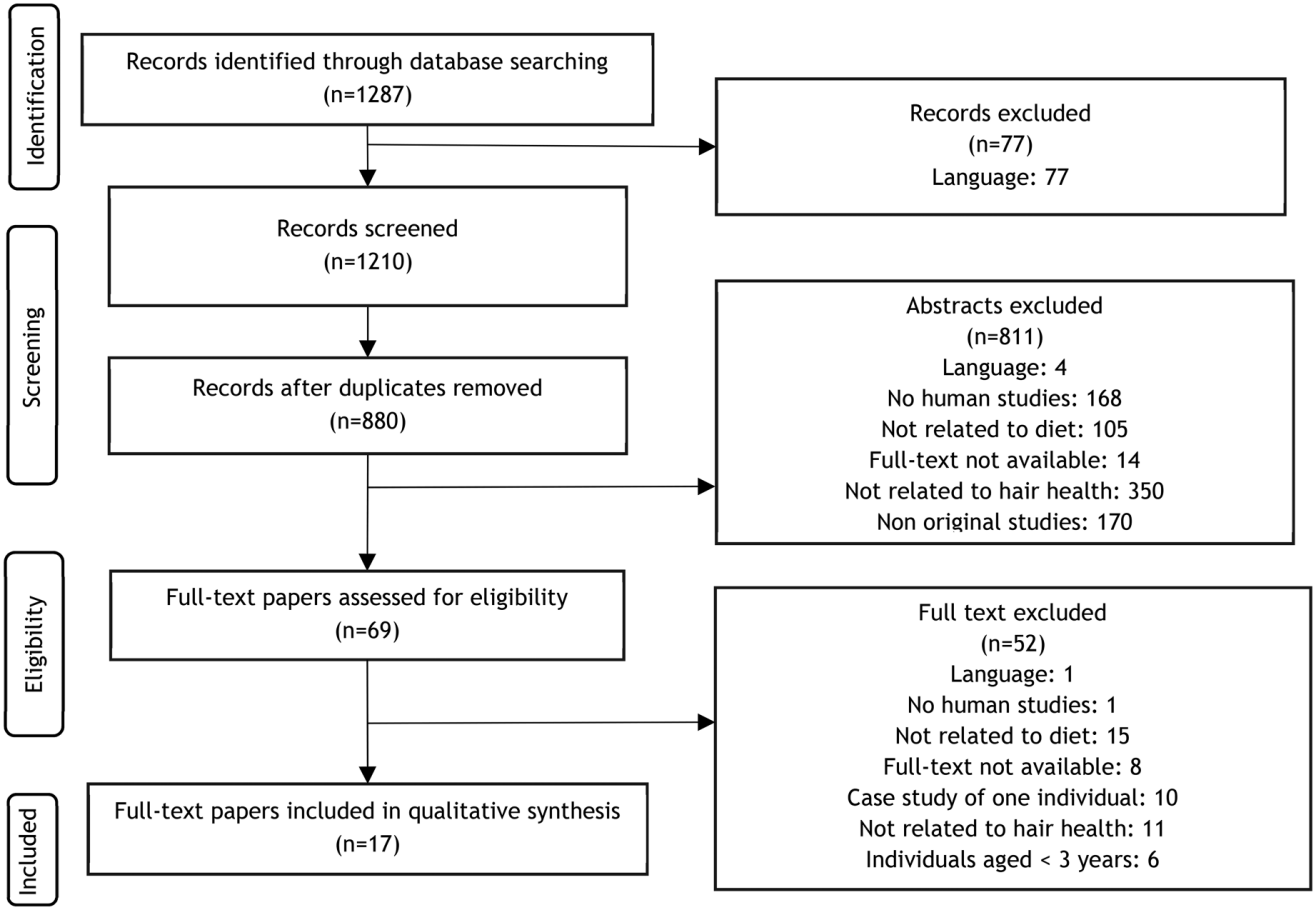
PRISMA fluxogram.

The number of studies excluded and the reasons for this are presented in the PRISMA flowchart, as illustrated in Figure 1.

It was not necessary to contact the authors, as all relevant data were already published. Duplicate articles were identified and removed using the Endnote tool (The EndNote Team, 2013). A total of 880 abstracts and 69 full articles were screened, with 17 studies ultimately included in this systematic review (Figure 1). The data from the included studies is presented in Table 2, categorized by author and year, country, study type, sample size, participant age and sex, dietary factor (food/nutrient), hair health determinant, and the respective association examined.

**Table 2.** Characteristics of the included studies evaluating the association between diet/nutrition and hair health.

Author, year (reference)	Country	Study type	Sample size	Age and sex	Dietary factor		Hair outcome		Association with hair health	Quality assessment score
					Nutrient	Food	Identification	Method		
Hagino T, et al. 2021 (Hagino et al., 2021)	Japan	Case-control 50% cases	*n* = 140	Mean, years Cases 47,5 Controls 49,0 73% women	Retinol	——	Alopecia Areata (AA)	Clinical history (National Alopecia Areata Foundation recommendations), Severity of Alopecia Score (SALT)	A high intake of retinol was found to be associated with a greater severity of AA.	5^a^
Kondrakhina IN, et al. 2021 (Kondrakhina et al., 2021)	Russia	Cross-sectional	*n* = 75	Mean (SD), years 26,2 (5,3) 100% men	Minerals magnesium, zinc, copper, iron, selenium Vitamins D, E, B_12_	——	Androgenetic alopecia (AGA)	Hamilton Norwood scale Clinical examination Trichogram Phototrichogram	The occurrence and progression of AGA have been linked to multiple micronutrient deficiencies, including those of zinc, copper, magnesium, selenium, vitamins B_12_, E, D and folic acid. Regarding copper, higher levels were associated with thicker hair in healthy individuals, while in patients with AGA, higher levels of copper were associated with thinner hair.	4^b^
Lin CS, et al. 2023 (Lin et al., 2023)	China	Cross-sectional	*n* = 155	Mean (SD), years 34,4 (12,7) 100% women	Iron ferritin <60 ng/mL 100 mg/cmp	——	Feminine alopecia	Previously diagnosed alopecia	Iron supplementation was associated with improvements in hair growth reported by the participants and an increase in ferritin levels.	6^b^
Bradfield RB, et al. 1971 (Bradfield, 1971)	USA	Clinical trial (2 weeks)	*n* = 6	21–35 years 100% men	Protein (0/12/24 g N/person)	——	Root: Diameter Pigmentation	Hair samples	Protein deprivation was associated with a reduction in the diameter (up to the 11th day) and pigmentation (up to the 14th day) of the hair bulbs.	High risk of bias^c^
Unal M, et al. 2018 (Unal and Gonulalan, 2018)	Turkey	Case-control (4 months) 37% cases	*n* = 54	Cases Mean (SD), years Boys: 12,4 (4,2) Girls: 13,3 (4,4) Controls Boys: 16,6 (0,8) Girls:16,5 (1,0) 46,2% women	Vit.D	——-	Alopecia Areata (AA)	Clinical history, Severity of Alopecia Score (SALT)	Serum vit.D levels correlated inversely with the severity and duration of AA.	6^a^
Sanke S, et al. 2020 (Sanke et al., 2020)	India	Case- control 50% cases	*n* = 100	Cases 23 years Controls 24,2 years 100% men	Vit.D	———	Premature Androgenetic Alopecia	Clinical history, Hamilton Norwood (I-VII) scale.	Serum vit.D levels were inversely correlated with the severity of premature AGA.	7^a^
Thompson JM, et al. 2016 (Thompson et al., 2016)	USA	Cohort (12 years) 0,24%	*n* = 55929	30–55 years 100% women	Vit.D	———	Alopecia Areata (AA)	Self-report	There was no association between vit D levels and the development of AA.	9^d^
Daroach M, et al. 2018 (Daroach et al., 2018)	India	Case-control (6 months) 50% cases	*n* = 60	Mean (SD), years Cases 28,9 (9,9) Controls 31,1 (9,4) 55% women	Vit.D	———	Alopecia areata (AA)	Clinical examination, including severity of alopecia score (SALT)	Serum vit.D levels correlated inversely with the severity and duration of AA.	6^a^
Aksu Cerman A, et al. 2014 (Aksu Cerman et al., 2014)	Turkey	Cross-sectional	*n* = 188	Mean (SD), years With AA 32,2 (9,6) Without AA 32,5 (9,7) 37,7% women	Vit.D	———	Alopecia Areata (AA)	Clinical history, Severity of Alopecia Score (SALT)	Serum vit.D levels correlated inversely with the severity of AA.	7^b^
Jacquet A, et al. 2007 (Jacquet et al., 2007)	France	Clinical trial (3 months)	*n* = 52	Mean, years 48,5 100% women	———	*INVERSION Femme* (green tea and grape extract, beta-carotene, Zn, Se, Cr, borage seed, shark cartilage, vitamins C, B_2_, B_5_, B_6_, B_8_, Cu, Fe, fish oil)	Hair loss	Consultations Measuring hair loss through brushing in individuals with identical haircuts Self-report	Consumption of INVERSION Femme was associated with less hair loss.	High risk of bias^c^
Kalman DS, et al. 2020 (Kalman and Hewlings, 2020)	USA	Randomized controlled trial (12 weeks)	*n* = 88	Mean (SD), years 53,3 (7,6) 71,6% women	———	Oral hydrolysed eggshell membrane (BiovaBio™) 450 mg/d	Density Breakage Growth	Computerized phototrichogram (TrichoScan HD)	Ingestion of hydrolysed oral eggshell membrane was associated with an increase in hair density, with no effect on hair breakage or growth.	High risk of bias^c^
Ham S, et al. 2023 (Ham et al., 2023)	South Korea	Randomized controlled trial (24 weeks)	*n* = 88	Mean (SD), years Patients 38,5 (8,0) Placebo 39,0 (8,8) 61,3% women	———	Persimmon leaf formulated with green tea and sophora fruit extracts	Density Thickness Gloss	Phototrichogram Hair count Gloss meter Scanning Electron Microscope (SEM)	Consumption of persimmon leaf was associated with hair density and thickness. There was also an increase in shine, although this was not statistically significant.	Medium risk of bias^c^
Ablon, G., 2015 (Ablon, 2015)	USA	Randomized clinical trial (3 months)	*n* = 60	Mean (SD), years 48,6 (10) 100% women	———	Oral marine protein supplement: AminoMar, Equisetum arvense sp. (horsetail which contains a natural form of silica), Malpighia glabra (vitamin C), biotin and zinc (1 cmp 2x/day).	Hair loss	Clinical examination Phototrichogram	Oral supplementation with proteins derived from marine sources promoted hair growth and reduced hair loss in women with temporary alopecia.	High risk of bias^c^
Nomura SJO, et al. 2018 (Nomura et al., 2018)	California	Cross-sectional	*n* = 365	Mean (SD), years 57,1 (10,4) 100% women	———	Soy-based products (≥24.0 g/d) Cruciferous vegetables (≥70.8 g/d)	Hair loss	Self-report	Higher soy intake was associated with less hair loss, although this was not statistically significant. Higher intake of cruciferous vegetables was associated with less hair loss.	7^b^
Akin Belli A, *et al*. 2016 (Akin Belli et al., 2016)	Turkey	Cross-sectional	*n* = 1119	Mean, years 17,4 66,7% women	———	Alcoholic beverages	Premature depigmentation	Self-report Clinical examination	The consumption of alcoholic beverages was associated with premature depigmentation of the hair.	6^b^
Yi Y, et al. 2020 (Yi et al., 2020)	China	Cross-sectional	*n* = 1825	18–40 years 100% women	———	Alcoholic beverages	Hair loss	Self-report, basic and specific scale (BASP)	The consumption of alcoholic beverages was associated with greater hair loss.	6^b^
Shi XJ, *et al*., 2023 (Shi et al., 2023)	China	Cross-sectional	*n* = 1028	Mean (SD), years 27,8 (7,2) 100% man	———	Sweet beverages (Average: > 3500 ml/week)	Hair loss	Self-report, basic and specific scale (BASP)	Excessive consumption of sweet beverages was positively related to hair loss.	8^b^

SD – Standard deviation.

^a^ The NOS for case–control studies (ranging from 0 to 9 stars).

^b^ The NOS for cross-sectional studies (ranging from 0 to 8 stars).

^c^ Tool of the Cochrane Collaboration for randomized control trials (low, medium, or high risk of bias), ^d^The NOS for cohort studies (ranging from 0 to 9 stars).

### Quality assessment

To assess the methodological quality of the studies included in this review, the Newcastle-Ottawa Scale (NOS) (Wells et al., 2000) was used for cohort and case-control studies, with a total score ranging from 0 to 9 points. A modified version of the NOS (Moskalewicz and Oremus, 2020) as used for cross-sectional studies, with a total score ranging from 0 to 8 points. This adapted version has been previously applied in the quality evaluation of other systematic reviews (Alshabanat et al., 2015; Modesti et al., 2016). Additionally, the Cochrane Collaboration's revised tool (Sterne et al., 2019) was used to evaluate the methodological quality and risk of bias of randomized controlled trials.

## Results

Figure 1 illustrates the process of study selection. This review included 17 articles, which are summarized in Table 2. The articles comprised seven cross-sectional studies, four case-control studies, three randomized clinical trials, two clinical trials and one cohort study. In total, the studies involved 613,320 individuals, 97% of whom were women (*n* = 594,800) with samples ranging in age from 7 to 77 years old (Lin et al., 2023). Nine articles were published between 1971 and 2018, while eight articles were published between 2020 and 2023. Regarding geographical distribution, 11 studies were conducted in Asia, followed by five in America (Ablon, 2015; Bradfield, 1971; Kalman and Hewlings, 2020; Nomura et al., 2018; Thompson et al., 2016) and one in Europe (Jacquet et al., 2007).

For quality assessment, the cross-sectional articles had a mean score of 6.29 (1.11) points (min: 4.00; max: 8.00), and the case-control studies included had a mean score of 6.00 (0.82) (min: 5.00; max: 7.00). The one cohort article had a score of 9.00 points. Considering the randomized control trials, one study presented a medium risk of bias and four articles a high risk of bias (Table 2).

The studies included in this review employed a variety of approaches to investigate the relationship between nutrients, foods and hair health outcomes. Nine studies focused on nutrients as the primary exposure factor, while eight investigated specific foods or supplements. Among the nutrient-focused studies, vitamin D was the most frequently examined (*n* = 5) (Aksu Cerman et al., 2014; Daroach et al., 2018; Sanke et al., 2020; Thompson et al., 2016; Unal and Gonulalan, 2018), followed by retinol, iron and protein, each assessed in a single study (Bradfield, 1971; Hagino et al., 2021; Lin et al., 2023). Among the food-related studies, four explored the effects of supplementation (Ablon, 2015; Ham et al., 2023; Jacquet et al., 2007; Kalman and Hewlings, 2020), two examined alcoholic beverages (Akin Belli et al., 2016; Yi et al., 2020), one assessed sugary drink consumption (Shi et al., 2023), and one investigated the consumption of cruciferous vegetables and soy products (Nomura et al., 2018).

The identified hair outcomes included the severity and occurrence of alopecia (*n* = 8 articles) and the assessment of hair parameters, such as density, growth, thickness, shine, and hair loss (*n* = 9 articles). Within alopecia research, studies investigated various types of the condition; five focused on alopecia areata (AA) (Aksu Cerman et al., 2014; Daroach et al., 2018; Hagino et al., 2021; Thompson et al., 2016; Unal and Gonulalan, 2018), one on androgenic alopecia (AGA) (Kondrakhina et al., 2021), one on premature AGA (Sanke et al., 2020), and one on female alopecia (Lin et al., 2023). Regarding alopecia assessment methodologies, clinical history was a prominent approach in four studies (Aksu Cerman et al., 2014; Hagino et al., 2021; Sanke et al., 2020; Unal and Gonulalan, 2018), while clinical examinations were used in two studies. The Severity of Alopecia Score (SALT) (Olsen, 2011) was applied in four studies (Aksu Cerman et al., 2014; Daroach et al., 2018; Hagino et al., 2021; Unal and Gonulalan, 2018).

Regarding hair parameters, hair loss was the most extensively studied, with five articles dedicated to this topic. The primary methodologies used for assessing these parameters were self-reporting (Akin Belli et al., 2016; Jacquet et al., 2007; Nomura et al., 2018; Shi et al., 2023; Yi et al., 2020) and phototrichograms (Ablon, 2015; Ham et al., 2023; Kalman and Hewlings, 2020).

### Nutrients

Regarding alopecia areata (AA), one study reported that high retinol intake was associated with increased disease severity (Hagino et al., 2021). Four studies investigated the potential benefits of vitamin D in AA. Three studies (Aksu Cerman et al., 2014; Daroach et al., 2018; Unal and Gonulalan, 2018) identified an inverse correlation between vitamin D levels and AA severity, while two studies (Daroach et al., 2018; Unal and Gonulalan, 2018) also found an inverse relationship between vitamin D levels and disease duration. However, one study found no association between vitamin D levels and AA development (Thompson et al., 2016).

Regarding androgenic alopecia (AA), one study found that deficiencies in multiple micronutrients (zinc, copper, magnesium, selenium, vitamins B_12_, E, D and folic acid) were associated with its occurrence and progression (Kondrakhina et al., 2021). Additionally, another study reported a correlation between serum vitamin D levels and the severity of AGA (Sanke et al., 2020).

Regarding hair health, iron supplementation (100 mg/tablet) was positively associated with improvements in hair growth (Lin et al., 2023). In contrast, a protein-deficient diet was linked to negative effects on hair health, including reduced hair bulb diameter and pigmentation (Bradfield, 1971).

### Foods

Regarding hair loss, the consumption of soy products (≥24.0 g/day) and cruciferous vegetables (≥70.8 g/day) was associated with a reduction in hair loss (Nomura et al., 2018). Two studies found that alcohol consumption negatively impacted hair health, contributing to increased hair loss (Yi et al., 2020) and premature depigmentation (Akin Belli et al., 2016). Additionally, one study reported that excessive consumption of sugary beverages was linked to a higher prevalence of hair loss (Shi et al., 2023). Two studies also showed that specific supplements, including INVERSION Femme and the oral marine protein supplement (one tablet, twice daily), were associated with reduced hair loss (Ablon, 2015; Jacquet et al., 2007). These supplements were also found to promote hair growth in women with temporary alopecia (Ablon, 2015).

Regarding hair density, one study investigated the effects of oral hydrolysed eggshell membrane supplementation (450 mg) and found a significant increase in hair density, without affecting other hair health parameters such as breakage or growth (Kalman and Hewlings, 2020). Furthermore, another study examined the effects of persimmon leaf consumption and reported that this food was associated with improvements in both hair density and thickness (Ham et al., 2023).

## Discussion

The 17 articles included in this systematic review offer a comprehensive overview of the relationship between food, nutrition and hair health. The primary nutrients and food items examined were vitamin D, iron, protein, soy, cruciferous vegetables, alcoholic, and sweet beverages, as well as specific supplements. These factors were most frequently linked to the severity and occurrence of alopecia and various hair parameters, including density, growth, thickness, shine, and hair loss. Overall, most of these factors showed a positive association with hair health, with the exceptions of alcoholic and sweet beverages, as well as retinol.

The systematic review included a total of 61,332 individuals, revealing a higher prevalence of alopecia in women compared to men. This finding aligns with the scientific literature, which reports that the prevalence of alopecia in women (11.4 million) is approximately twice that observed in men (6.1 million) (IHME). The broad age range in this review (7 to 77 years old) demonstrates that alopecia affects individuals across various age groups. The Institute for Health Metrics and Evaluation (IHME) reports that in 2021, 0.38% of individuals aged 30–34, 0.36% of those aged 35–39, and 0.31% of those aged 24–29 worldwide had alopecia (IHME, 2024). These statistics are consistent with the findings of this review, as nine of the 17 included articles focus on individuals within this age group. The review also includes one article on pediatric alopecia (Lin et al., 2023), and two studies on adolescents. Despite alopecia being relatively common in children, the incidence and prevalence in the pediatric population remain unclear (Griggs et al., 2021). Given the limited evidence in the existing literature, further research is needed to confirm these findings.

Androgenic alopecia (AGA) is the most prevalent form of alopecia, affecting approximately 50% of men by the age of 50, and it is also common in women, particularly after menopause (Ho et al., 2024). A relationship between AGA and diet has been established in the literature (Almohanna et al., 2019; Rajput, 2022). The two articles included in this review align with previous studies, indicating that nutritional deficiencies may contribute to the development of AGA. Although alopecia areata (AA) it's not the most prevalent form of alopecia globally, its autoimmune nature has sparked significant clinical and scientific interest in understanding its underlying mechanisms and exploring potential therapeutic approaches (Bertolini et al., 2020; Paus, 2020). This explains the greater number of studies on AA identified in this review compared to those on AGA.

Although accurately determining the prevalence of other forms of alopecia is challenging, it is well-established that these conditions affect a portion of the population in response to stress, illness, or other factors (Kesika et al., 2023; Shimizu et al., 2022). One such condition is telogen effluvium, characterized by diffuse, often acute hair loss, which can be linked to diet, particularly in cases of iron deficiency or excessive vitamin A intake (Hughes et al., 2024). Two studies included in this review support these findings, indicating that these nutrients influence hair loss (Hagino et al., 2021; Lin et al., 2023). It is estimated that at least 3.3 billion people worldwide may experience some form of hair loss (IHME, 2024; Nations, 2022), predominantly due to AGA. This figure may even be an underestimate, as it does not account for other forms of alopecia or temporary hair loss (Ho et al., 2024; Shimizu et al., 2022).

It is noteworthy that the outcomes related to hair health in the studies included in this review varied depending on the exposure being analysed. Specifically, studies that focused on the impact of individual nutrients on alopecia primarily investigated this condition, while those examining food consumption looked at their influence on hair loss and hair density. This distinction can be attributed to the involvement of different mechanisms. The effects of single nutrients on hair health can be direct and specific (Chen et al., 2018). For example, iron supplementation has been associated with hair growth and the prevention of hair loss (Trost et al., 2006). In contrast, whole foods contain a combination of nutrients that can interact in a more complex ways, affecting hair loss and density in a more generalized way. Additionally, the nutrient-specific approach allows for more precise control over exposure factors in studies, whereas the analysis of whole foods takes into account the combined effects of several nutrients and bioactive compounds (Braun and Heinrich, 2020).

The systematic review revealed that vitamin D was the most studied nutrient, featured in five out of the nine articles on nutrients. In general, vitamin D was found to be inversely correlated with the severity of AA and AGA, which aligns with previous studies highlighting the association between vitamin D deficiency and both conditions (Amor et al., 2010; Saini and Mysore, 2021; Zhao et al., 2020; Zubair et al., 2021). The data suggests that the Vitamin D Receptor (VDR) plays a significant role in the hair follicle cycle, by regulating the initiation of the anagen phase through ligand-independent mechanisms and by interacting with the Wnt/β-catenin signalling pathway and the nuclear receptor Hairless (Hr) to maintain proper hair follicle homeostasis. This highlights the VDR's crucial involvement in transcriptional regulation that activates hair growth and sustains follicle stem cell populations, underscoring the need for further research into the role of vitamin D and the VDR in the hair cycle to address this issue effectively (Amor et al., 2010). In this context, Seleit et al. (2020) demonstrated that polymorphisms of these receptors (Taq-1 and Cdx-1) influence hair loss and can serve as risk factors for the duration of the disease (Seleit et al., 2020).

Regarding iron, the present review suggests that iron supplementation is beneficial, with reported improvements in hair growth. A review by Trost et al. (2006) noted that several studies have positively linked iron deficiency to hair loss, particularly in women, which can be explained by iron's crucial role in hair follicle metabolism as a cofactor for enzymes involved in DNA synthesis and cellular proliferation – processes essential for the rapidly dividing cells in hair follicles. In that review, some studies proposed a correlation between iron deficiency and both AA and AGA, while others refuted this hypothesis, concluding that the evidence was insufficient to recommend iron supplementation for patients with alopecia (Trost et al., 2006). Further research is required to deepen our understanding of the relationship between iron supplementation and its impact on hair health.

The review included a study that found that protein deprivation was associated with a reduction in the diameter and pigmentation of hair bulbs. This occurs because hair shaft is primarily composed of keratin, a structural protein that requires adequate amino acid availability for proper synthesis and hair follicle function (Bradfield, 1971). These findings align with the conclusions of the review by Guo and Katta (2017), which indicated that protein malnutrition, as seen conditions like kwashiorkor and marasmus, can lead to hair changes, including hair thinning and hair loss. Furthermore, the same review highlighted a study on L-lysine, an essential amino acid involved in the absorption of iron and zinc. The combination of iron supplementation with L-lysine resulted in a significant increase in mean serum ferritin concentration in some women with chronic hair loss who had shown an inadequate response to iron supplementation alone (Guo and Katta, 2017).

The findings of this systematic review suggest a correlation between alcohol consumption and an increased incidence of hair loss, as well as a potential inhibitory effect on hair growth (Yi et al., 2020). The relationship between alcohol consumption and alopecia areata is complex, as evidenced by the literature. Alcohol has been shown to relieve psychological stress (Minokawa et al., 2022; Wang et al., 2019), which is an important factor in the development of alopecia areata (Gupta et al., 1997). In this context, the study by Dai et al. (2020) found that social and regular drinkers had a significantly lower risk of developing AA compared to those who had never consumed alcohol (Dai et al., 2020). However, the current body of literature does not allow for a definitive conclusion about the impact of alcohol consumption on alopecia. Further research is needed to clarify this relationship and better understand the underlying mechanisms.

Regarding the consumption of foods with a high sugar content, one of the included articles indicated that sugary drink consumption is associated with an increased risk of hair loss in young males (Shi et al., 2023). This finding is supported by Goluch-Koniuszy (2016), who reported that the consumption of processed foods containing simple sugars is an indirect factor linked to hair loss. These foods stimulate the secretion of excess sebum, which promotes microbial growth on the scalp. This, in turn, exacerbates irritation and inflammation, contributing to hair loss (Goluch-Koniuszy, 2016).

In this review, one article indicated that a higher intake of soy products and cruciferous vegetables was associated with a reduction in hair loss. This finding is consistent with the work of Pham et al. (2020), which showed that diets rich in vegetables and soy products, particularly those containing isoflavones, promote hair health and growth. Additionally, the phytochemicals found in these foods, such as carotenoids and polyphenols, possess anti-inflammatory and antioxidant properties. These properties help reduce the production of reactive oxygen species in dermal papilla cells, leading to decreased secretion of transforming growth factor β1 and increased hair growth stimulation (Pham et al., 2020). However, further research is necessary to deepen our understanding of the role of these foods in hair health.

In the context of complex dietary supplements, four articles have addressed this subject, and all have yielded evidence of improvements in hair parameters (Ablon, 2015; Ham et al., 2023; Jacquet et al., 2007; Kalman and Hewlings, 2020). The synergistic effects of multiple nutrients in these formulations may address various aspects of hair follicle metabolism simultaneously, from providing structural building blocks to supporting cellular energy production and antioxidant defence mechanisms. A review by Braun and Heinrich (2020) corroborates this data, stating that certain complex dietary supplements can improve hair loss and emphasizing the importance of a multifaceted approach to treating certain hair parameters (Braun and Heinrich, 2020).

The main limitations of this systematic review are that most of the studies are cross-sectional in design, which prevents the establishment of cause-and-effect relationships between the observed associations. Furthermore, the heterogeneity of the studies, including variations in age groups, food/nutrient studied, health outcomes and assessment methods, should be considered. Due to this heterogeneity and the limited number of articles included in this review, it was not possible to conduct a meta-analysis. Therefore, further studies are needed to confirm and strengthen the data presented in this review.

This study is the first systematic review to explore the impact of nutrients and foods on hair health. Adherence to the PRISMA methodology ensured a thorough and systematic assessment of the data. In terms of public health, this review makes a significant contribution to the existing body of knowledge, offering insights that can help and inform the practices of healthcare professionals in this area. As the demand for the latest evidence-based information on the safety, tolerability, and efficacy of nutritional therapies for hair loss grows, this review provides valuable guidance for both patients and healthcare providers.

## Conclusion

The findings of this review highlight the significant role that dietary and nutritional factors play in maintaining hair health. The results suggest that the intake of specific nutrients and foods may influence hair health. Higher serum vitamin D levels and iron supplementation were found to positively impact alopecia, reducing its severity and promoting hair growth, respectively. A positive association was also observed between protein intake, the consumption of soy products, cruciferous vegetables and supplements, with improvements in hair parameters such as hair loss and hair density. On the other hand, a higher consumption of alcoholic and sugary drinks was associated with an increased risk of hair loss. The findings suggested that targeted dietary interventions could be important in preventing and managing hair conditions such as alopecia and hair loss. However, further research is needed to deepen our understanding of these associations and to develop evidence-based recommendations for promoting hair health through nutrition.
